# Prescribing of medication for attention deficit hyperactivity disorder among young people in the Clinical Practice Research Datalink 2005–2013: analysis of time to cessation

**DOI:** 10.1007/s00787-017-1011-1

**Published:** 2017-06-06

**Authors:** Tamsin Newlove-Delgado, Tamsin J. Ford, William Hamilton, Ken Stein, Obioha C. Ukoumunne

**Affiliations:** 1 University of Exeter Medical School, St Luke’s Campus, Heavitree Road, Exeter, EX1 2LU UK; 2 NIHR CLAHRC South West Peninsula (PenCLAHRC), University of Exeter Medical School, St Luke’s Campus, Heavitree Road, Exeter, EX1 2LU UK

**Keywords:** ADHD, Psychopharmacology, Prescribing, Discontinuation, Transition

## Abstract

The aim of this study was to examine the time to cessation of ADHD medication amongst young people with ADHD aged 16 in the period 2005–2013. Previous studies of prescribing in primary care reported high rates of medication cessation amongst 16 and 17 year olds with ADHD. The examination of trends since the introduction of new NICE guidance in 2008 will support service planning and improvement of outcomes over the vulnerable transition period from child to adult services. We used primary care records from the Clinical Practice Research Datalink and identified cases prescribed ADHD medication at the time of their 16th birthday during the study period. The outcome was time to medication cessation from the age of 16. Cessation of medication was defined as occurring at the beginning of a gap of over 6 months in prescriptions. 1620 cases were included. The median time to cessation was 1.51 years (95% CI 1.42–1.67).The estimated probability of remaining on medication was 0.63 (95% CI 0.61–0.65) at age 17 (i.e., at 1 year) and 0.41 (95% CI 0.39–0.43) at age 18. Young people with ADHD remain at high risk of cessation of medication during the transition from child to adult services. Despite the restriction that only primary care prescribing data were available, the results suggest continuing disparity between expected levels of symptom persistence and continuation of medication.

**Electronic supplementary material:**

The online version of this article (doi:10.1007/s00787-017-1011-1) contains supplementary material, which is available to authorized users.

## Introduction

The majority of young people with ADHD are likely to experience the persistence of symptoms beyond the age of 16. Faraone and colleagues’ review reported that up to 65% of those with the condition were likely to remain symptomatic at age 25, although only 15% met full diagnostic criteria at this age [1]. A recent systematic review of persistence suggested that 40–50% still met criteria for a diagnosis in adulthood where recommended methods such as age-appropriate symptom thresholds were used [2]. In contrast, studies of prescribing practices across Europe, the US and the UK report high rates of medication discontinuation in late adolescence, at a steeper and more rapid rate than would be expected given the rate of decline in symptoms reported by longitudinal studies [3–6].

In the UK, adolescents with ADHD are managed by child and adolescent mental health services or by paediatric services. Specialists in these clinics will initiate medication if required, but ongoing prescribing is usually carried out by the General Practitioner (GP) in primary care, under shared care arrangements. Young people move on from child services between the ages of 16 and 18, but historically there have often not been adult services available to those with ADHD to support ongoing prescribing. However, no UK study has to date examined prescribing over the transition period using data collected since the introduction of the UK National Institute for Health and Care Excellence (NICE) guidance in 2008, which first recommended continuation of treatment for ADHD into adulthood if indicated [7]. Earlier studies of ADHD prescribing using primary care databases in the UK have covered the periods 1999–2006 in the Clinical Practice Research Datalink [5] and 2003–2008 in the Health Improvement Network [6]. Over time, there has been increasing recognition of the challenges involved in the transition from child to adult services for young people with ADHD, which may include a lack of commissioned services, shared care arrangements and care pathways, and potentially the attitudes and knowledge of clinicians [8–13]. The ‘drop-off’ in prescribing seen in previous studies in late adolescence may, therefore, relate partially to these barriers to ongoing treatment over the transition period, as well as to patient choice and/or clinical judgement. To better understand recent patterns and practice in prescribing for young people with ADHD in transition, we used the most recently available data on primary care prescribing in the Clinical Practice Research Datalink (from 2005 until the end of 2013) to study prescribing and describe the distribution of the time to cessation in young people with ADHD. We also examine factors which might be associated with cessation of medication.

## Methods

### Study design

We carried out a survival analysis of time to cessation of ADHD medication prescribed in primary care from the age of 16 in a cohort of patients from the Clinical Practice Research Datalink (CPRD). This age was chosen as it marks the beginning of the formal transition period in the UK from education and from children’s health services to adult provision, and would capture the period where cessation rates were highest in previous studies [5, 6]. The CPRD is a large UK clinical database run by the Medicines and Healthcare products Regulatory Agency (MHRA). The primary care section contains the records of over 11 million patients and covers up to 6% of the population [14]. CPRD extracts anonymised data from participating GP practices’ IT systems. All prescriptions issued in primary care are automatically captured. Cases included in a research dataset must have a minimum duration of ‘up-to-standard’ data as defined by a metric examining continuity of recording by a practice and recording of events in a patient record. All protocols using patient-level data from the CPRD are reviewed and approved by the Independent Scientific Advisory Committee (ISAC) on behalf of the National Research Ethics Service Committee. This study protocol (13_213) was granted approval in 2013.

### Patients

We obtained a dataset from CPRD containing the records of all patients who were aged between 10 and 20 in 2005, and had a diagnosis of ADHD coded in their file and/or at least one prescription for an ADHD medication. Both were defined using a list of codes in the study protocol (available as supplementary material).

From the cohort (*n* = 9390), a subset of patients (*n* = 1620) met the criteria for the survival analysis, which were:

- At least 6 months of consecutive prescriptions for an ADHD medication for the time period up to and including the 1st July of the year of their 16th birthday. A gap of up to 6 months between prescriptions was allowed.
- At least 1 day of follow-up data in the CPRD following their 16th birthday.

Patients were excluded if they had a gap in their registration with the practice for more than 6 months after their 16th birthday, meaning that follow-up data were not available. As CPRD does not provide the full date of birth, this was designated to be 1st July for each case, which minimised the error each way to a maximum of 6 months. As transition is a process, rather than a single event happening on a birthday, this did not affect the ability of the study to encompass the relevant time period of transition. The study period ran from 1st Jan 2005 until 31st Dec 2013.

### Analysis

Stata version 13 was used to perform the survival analysis [15].The entry point was the 1st July in the year the patient turned 16. The observation period (time at risk) was then from this date until cessation or censoring (i.e., for as long as data were recorded for that patient) or the study period ended (31st Dec 2013). Cessation was defined as the beginning of a gap of more than 6 months in prescriptions for ADHD medication. In some cases, patients left practices which contribute data to the CPRD and, therefore, their records were no longer included. Censoring occurred in the dataset where there were no further prescribing records for a case within the study period, and the outcome i.e., whether and at what point cessation occurred, was unknown. Cases that were censored, therefore, are those who were still being prescribed medication up until the point of leaving a CPRD practice and being lost to follow-up. This was defined as having a prescription dated within 40 days of the date of being lost to follow-up through the CPRD.

We summarised the distribution of time to cessation using the Kaplan–Meier estimator. We fitted Cox regression models to explore variables recorded in CPRD which might predict cessation of ADHD medication (gender, prescription for another psychotropic medication at age 16 or over, referral to adult psychiatry at any point, diagnoses of anxiety or depression, conduct/oppositional defiant disorder or autistic spectrum disorder (ASD) at any point, learning disability, year of birth, smoking at any time, and time on ADHD medication before 16th birthday).

Each putative predictor variable was explored singly in crude (unadjusted) Cox regression models, reporting hazard ratios. All predictors with a *p* value less than 0.05 were then entered into a multivariable (adjusted) Cox regression model. We tested the proportional hazards assumption for each model by examining Schoenfeld residuals and plotting Nelson–Aalen cumulative hazard estimates [16].

Sensitivity analyses were also carried out to assess the effect of excluding the cases censored before the end of the study from the analysis, and of extending the definition of censoring to include cases that had a prescription within 90 days of leaving a CPRD practice.

Estimates from CRPD in 2012 prior to obtaining the dataset were that there would be approximately 1100 eligible individuals, with 80% having follow-up data available for 8 or more years. Our power calculations suggested this sample size would be large enough to estimate the percentage of the cohort that remains on medication in adulthood with a margin error of no greater than ±3.3 percentage points based on the width of the 95% confidence interval.

## Results

### Sample

The analyses included 1620 patients who met the criteria for entry, of which 1419 (87.6%) were male. The majority of cases (*n* = 1230; 75.9%) experienced cessation during the follow-up period. By the end of the study period, 302 patients were still prescribed medication and the remaining 88 were censored (i.e., they were lost to follow-up whilst still prescribed medication). The censored cases were similar to the uncensored cases with respect to starting time, duration of medication and year of birth.

### Cessation of medication

**Fig. 1:**
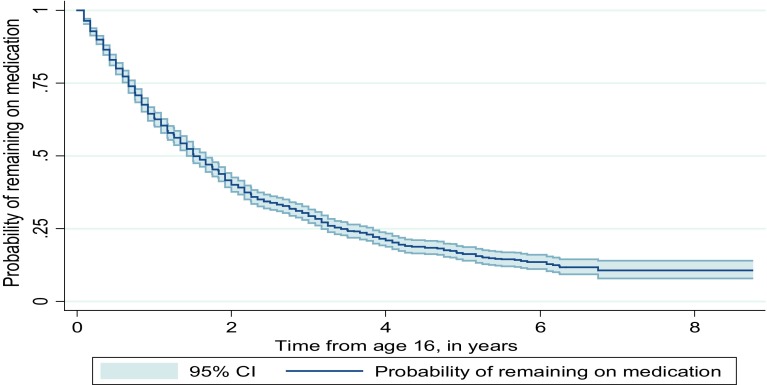
The probability of remaining on ADHD medication over time for young people prescribed medication at age 16 (Kaplan–Meier plot, *shaded area* represents 95% confidence intervals)

The median time to cessation was 1.5 years (95% CI 1.4–1.7), with the interquartile range of 0.7–3.4 years. A Kaplan–Meier plot (Fig. 1) displays survival probabilities, which in this case is the cumulative probability of an individual remaining on medication (i.e., not experiencing the event of cessation) at any time after baseline. Table 1 displays the probability of remaining on medication at 1 year time point from the age of 16. At age 17, the probability of remaining on medication was 0.63 (95% CI 0.61–0.65); at age 18 this was 0.41 (95% CI 0.39–0.43). By age 19 years this had fallen further to 0.30 (95% CI 0.28–0.32). At age 22 years, only 39 cases remained on medication in the study, the rest were censored by reaching the end of the study period, by being no longer registered with a CPRD contributor practice, or by experiencing cessation.

**Table 1:** The cumulative probability of remaining on ADHD medication over time for cases prescribed ADHD medication at the age of 16

Time from 16th birthday in years (age)	*n* with a prescription for ADHD medication	*n* stopping ADHD medication	Probability of remaining on ADHD medication (also known as the survival function) (95% CI)
0 (16 years)	1620	–	1
1 (17 years)	1016	590	0.63 (0.61–0.65)
2 (18 years)	646	343	0.41 (0.39–0.43)
3 (19 years)	337	159	0.30 (0.28–0.32)
4 (20 years)	165	91	0.21 (0.19–0.23)
5 (21 years)	75	30	0.16 (0.14–0.19)
6 (22 years)	39	11	0.13 (0.10–0.16)
7 (23 years)	11	6	0.11 (0.08–0.14)
8 (24 years)	11	0	0.11 (0.08–0.14)

### Variables associated with cessation

The results of the multivariable Cox regression analysis are presented in Table 2 below. Having a learning disability, an ASD diagnosis, and being referred to adult psychiatry at any point were associated with a lower likelihood of cessation, with a hazard ratio (HR) for cessation of 0.60 (95% CI 0.47–0.77), 0.68 (95% CI 0.55–0.83) and 0.67 (95% CI 0.54–0.83), respectively. Cases with prescriptions of psychotropic medication at the age of 16 or were also less likely to experience cessation than those who had no such prescription recorded (HR 0.79, 95% CI 0.67–0.91). Having an ADHD prescription for 3 or more years prior to the age of 16 was also associated with a reduced likelihood of cessation of medication. Those born later in the cohort were marginally less likely to experience cessation compared with those born earlier between 1989 and 1992 (HR 0.88, 95% CI 0.77–1.00), even after adjusting for all other variables (*p* = 0.05).

**Table 2:** Factors associated with the probability of cessation of ADHD medication from age 16 onwards (taken from a fully adjusted multivariate Cox regression model)

Variable	Hazard ratio (the probability of experiencing cessation for those with this characteristic compared to those without)	95% confidence interval for hazard ratio
Other psychotropic prescription aged 16 or over	0.79	0.67–0.91
Autism Spectrum Disorder	0.68	0.55–0.83
Learning disability	0.60	0.47–0.77
Referral to adult psychiatry	0.67	0.54–0.83
Smoking (ever recorded)	1.10	0.98–1.24
Birth year 1993–1995 (vs 1989–1992)	0.88	0.77–1.00
Time on medication prior to 16th birthday		
Less than 2 years	Reference	Reference
2–3 years	1.04	0.89–1.21
3 or more years	0.81	0.70–0.94

The *p* value for the global test of non-proportionality was 0.55, which suggested that the assumption of proportional hazards was reasonable. The planned sensitivity analyses to assess the effect of excluding the cases censored before the end of the study, and of extending the definition of censoring, did not appreciably alter the estimates obtained, and did not improve the fit of the model (details available on request).

## Discussion

### Time to cessation

In our sample, the majority of participants prescribed medication at the age of 16 had stopped taking it 2 years later. The rate of cessation that we report remains greater than the estimated rate of decline of symptoms from epidemiological studies. Faraone et al.’s meta-analysis found the persistence of ADHD symptoms meeting the full criteria for the condition 1 year later to be 83%, whereas the probability of medication persistence in our study was 63%. Faraone et al. place the persistence of symptomatic ADHD at age 20 at 69%, and a more conservative estimate of persistence of the full ADHD syndrome at 28%; both in excess of the probability of remaining on medication by age 20 in this study (21%) [1].

In contrast, Beau-Lejdstrom and colleagues analysed UK ADHD prescribing in under-16 s between 1992 and 2013 and found that more than three-quarters of children were still taking medication after 1 year and 60% were still on medication after 2 years [17]. Our findings are more similar to estimates from McCarthy et al.’s earlier study where approximately 40% of those starting medication in childhood or adolescence remained on medication at age 18 [6].

However, there is some evidence that prescribing patterns may be changing. Whilst a direct comparison should not be made with earlier studies, the populations are similar enough to note that our estimates of the rate of cessation from this analysis are slightly lower than those reported in the CADDY study, which covered the period from 1999 to 2006 [5]. Furthermore, those born later in our cohort who reached the age of 16 between 2009 and 2011 had a reduced hazard of cessation compared with those born earlier, with the lowest likelihood of cessation amongst those turning 16 in 2011. These findings may reflect changing prescribing practice in the UK over the transition period, the development of adult services and an increasing acceptance that ADHD continues to merit pharmacological intervention after the age of 16, possibly influenced by guidance from NICE in 2008 and the British Association for Psychopharmacology [7, 18].

### Predictors of cessation

Being referred to adult psychiatry at any point was associated with a markedly reduced likelihood of medication cessation. This may reflect a greater willingness of GPs to continue prescribing ADHD medication with the specialist oversight recommended by guidelines [18]. It is also likely that such patients would have more severe or persistent ADHD symptoms, which would mean they were both more likely to be referred and to continue medication.

The prescription of other non-ADHD psychotropic medication at the age of 16 or over was also associated with a reduced likelihood of medication cessation. This factor may be a marker for severity of ADHD or for other patient factors influencing help seeking behaviour and engagement with services [19]. We did not find an association between cessation and gender (unadjusted HR for females of 0.93; 95% CI 0.78–1.10), in contrast to the CADDY study [5]. This finding may have been influenced by the younger starting age in CADDY, or by secular changes. The gender distribution in our sample was in line with that from other studies of ADHD prescribing—12% of our sample were female, compared to 15% in Beau-Lejdstrom’s recent study [17] and 9% in the older CADDY study [5].

The only comorbid psychiatric diagnosis associated with a lower likelihood of cessation was ASD. Explanations for this finding are varied; people with ASD may be more likely to adhere to medication, or to experience greater impairment [20]. Due to their dual neurodevelopmental disorders they may also be managed in services such as paediatrics or learning disability, where transition may occur later or where there may be a greater acceptance of the use of medication. A similar explanation could apply to the association between learning disability and remaining on ADHD medication.

### Strengths and limitations

The chief strength of this analysis is the use of high quality and recent data from a national database capturing primary care prescribing until the end of 2013. The dataset covered a period which included the introduction of the NICE guidance on prescribing for adults and an expansion in awareness of adult ADHD. Use of shared care protocols means that in the UK, GPs undertake the majority of prescribing in ADHD, with specialist oversight [21]. Consequently, primary care prescriptions are likely to provide the fullest available picture of prescribing without using data linkages. Alternative sources such as dispensing records may offer more limited details on the diagnoses of the patient and other prescriptions which may be issued to them [10].

Nonetheless, primary care records will not capture prescriptions issued in specialist services which are not then passed on to primary care to continue, for example, in shorter-term trials of medication, or highly severe and complex cases. Where prescriptions are initiated outside primary care then transferred over to the GP, the length of time that an individual has been on medication may be underestimated, although the date of cessation (which was central to this analysis) would be unaffected. It is also important to consider that the outcome in this study was cessation of prescribing; some young people may not have been adherent to their medication and may have stopped taking it at an earlier date, if they took it at all [22]. With no standard definition of how long without prescribing constitutes ‘cessation’ of medication, we also had to decide upon a definition of cessation; the choice of a minimum 6-month period without prescribed medication was taken to allow for errors in estimating prescription length, medication breaks, and administrative delays.

There were fewer than 200 individuals remaining in the survival analysis after 4 years of follow-up, with greater uncertainty around the probability of cessation beyond the age of 20. Subjects censored during the study period may also have affected the results if the censoring was informative. This could apply to individuals whose registration with a CPRD practice was terminated before the end of the study period and who were prescribed medication until the point that their registration finished. This termination of registration could be related to moving away, which feasibly could be related to stopping medication, if it is not continued elsewhere. Alternatively, it could be due to moving to higher education and indicate a propensity to continue medication for the purpose of studying. Nonetheless, when these subjects were removed in a sensitivity analysis, there was no significant effect on the findings, suggesting minimal influence of informative censoring.

The main limitation of using this primary care dataset was the lack of clinical detail coded in the records, such as measures of ADHD symptoms and severity. It was not possible to determine for each case whether cessation took place due to remission of ADHD, patient choice, or ineffectiveness of medication; or whether it was due to service-related factors. Instead, the results should be considered in the light of other research into transition to provide context and explanations for exploration in future studies.

### Implications

Despite the existence of guidance on the management of ADHD in young people in transition, research findings imply that these recommendations are not always implemented in UK clinical practice. Hall and colleagues report wide variation in the provision of services and in prescribing arrangements for people with ADHD in transition in England [10, 11]. Professionals also report a lack of confidence in prescribing for ADHD in over-18s [9, 23, 24]. Given these potential barriers to ongoing prescribing, it is, therefore, plausible that a proportion of the young people in our study may be stopping medication from which they could still benefit, and may, therefore, be at greater risk of experiencing the adverse outcomes associated with ADHD [25].

The experiences of clinicians including paediatricians, psychiatrists and GPs are important both in understanding how these results reflect what is happening in everyday practice; and in considering how to target barriers and facilitators to optimising ADHD management in primary care and specialist services. Beliefs and knowledge will be influential in determining whether and how commissioners and clinicians implement the guidance on managing young people with ADHD in transition and, therefore, warrant further investigation through interview or survey studies.

Prescribing for young adults will also be heavily influenced by transition pathways and by the availability of services where ADHD medication can be monitored. There are added complications due to the UK prescribing regulations on controlled ADHD drugs, which may be implemented differently across localities and create further complexity in commissioning and in shared care arrangements [18]. Furthermore, prescribing is only one part of the approach to managing young people with persisting ADHD symptoms. There are various models of delivering care for older adolescents and adults with ADHD including extension of child mental health services, youth services and specific adult ADHD services, and recent recommendations on transition have been made by the UK Adult ADHD Network (UKAAN) [26–28]. However, the evidence regarding the effectiveness of different service models in improving outcomes needs to be strengthened—a systematic review in 2015 by Paul and colleagues concluded that there was currently no ‘high quality’ evidence to support the use of any particular transition care models [29]. Studies of adherence suggest that young adults may view stopping medication as an exertion of their autonomy, or may perceive that the negatives of medication outweigh the positive [22]. Consequently, there is a related question to be answered around what interventions or delivery models are most acceptable to young people, and would increase their engagement with managing their ADHD, and with services at this vulnerable time.

In conclusion, we detected high levels of cessation of prescribing for young people reaching transition age, which in combination with epidemiological data suggest that some young people may be stopping pharmacological treatment when they could still benefit from it. Robust evaluation of the cost-effectiveness, outcomes and acceptability of different service configurations is, therefore, necessary and would support and inform investment by commissioners in an environment where resources are scarce.

## Electronic supplementary material

Below is the link to the electronic supplementary material.

Supplementary material 1 (DOCX 18 kb)
