# Supplementary material for: Prescribing of medication for attention deficit hyperactivity disorder among young people in the Clinical Practice Research Datalink 2005–2013: analysis of time to cessation
Source: Eur Child Adolesc Psychiatry. 2017 Jun 6;27(1):29–35. doi: 10.1007/s00787-017-1011-1 (PMC5799339; doi:10.1007/s00787-017-1011-1)
Supplement: Supplementary file 1 — Supplementary material 1 (DOCX 18 kb) [file 787_2017_1011_MOESM1_ESM.docx]

Prescribing of medication for Attention Deficit Hyperactivity Disorder among young people in the Clinical Practice Research Datalink 2005-2013: analysis of time to cessation

Supplementary Material

**Table A1: List of Clinical Practice Research Datalink (CPRD) diagnostic codes eligible for inclusion in the analysis**

| **CPRD medical code** | **Read code** | **Read term** |
| --- | --- | --- |
| 6519 | Eu90011 | [X]Attention deficit hyperactivity disorder |
| 5565 | E2E0.00 | Child attention deficit disorder |
| 26285 | Eu9y700 | [X]Attention deficit disorder |
| 9715 | E2E0100 | Attention deficit with hyperactivity |
| 101067 | 6A61.00 | Attention deficit hyperactivity disorder annual review |
| 24808 | ZS91.11 | ADD - Attention deficit disorder |
| 28543 | ZS91.00 | Attention deficit disorder |
| 1458 | Eu90.00 | [X]Hyperkinetic disorders |
| 24546 | Ry13.00 | [D]Overactivity |
| 20467 | E2E0z00 | Child attention deficit disorder NOS |
| 45263 | E2E2.00 | Hyperkinetic conduct disorder |
| 24753 | ZS91.12 | [X]Attention deficit disorder |
| 33505 | Eu90100 | [X]Hyperkinetic conduct disorder |
| 37994 | ZS9..00 | Disorders of attention and motor control |
| 34199 | E2E0000 | Attention deficit without hyperactivity |
| 50015 | Eu90z00 | [X]Hyperkinetic disorder, unspecified |
| 41769 | E2Ez.00 | Hyperkinetic syndrome NOS |
| 37894 | ZS94.00 | Minimal brain dysfunction |
| 58069 | E2E1.00 | Hyperkinesis with developmental delay |
| 45799 | Eu90111 | [X]Hyperkinetic disorder associated with conduct disorder |
| 6510 | Eu90y00 | [X]Other hyperkinetic disorders |
| 96770 | Eu90z12 | [X]Hyperkinetic syndrome NOS |

**Table A2: List of Clinical Practice Research Datalink (CPRD) ADHD medication codes eligible for inclusion in the analysis**

| CPRD product code | CPRD product name |
| --- | --- |
| 5811 | Concerta XL 36mg tablets (Janssen-Cilag Ltd) |
| 576 | Methylphenidate 10mg tablets |
| 5810 | Concerta XL 18mg tablets (Janssen-Cilag Ltd) |
| 2679 | Ritalin 10mg tablets (Novartis Pharmaceuticals UK Ltd) |
| 7101 | Methylphenidate 5mg tablets |
| 9738 | Dexamfetamine 5mg tablets |
| 6169 | Methylphenidate 36mg modified-release tablets |
| 35159 | Concerta XL 27mg tablets (Janssen-Cilag Ltd) |
| 6868 | Equasym XL 20mg capsules (Shire Pharmaceuticals Ltd) |
| 6107 | Methylphenidate 18mg modified-release tablets |
| 14331 | Equasym XL 30mg capsules (Shire Pharmaceuticals Ltd) |
| 14512 | Dexedrine 5mg tablets (Auden McKenzie (Pharma Division) Ltd) |
| 14346 | Equasym XL 10mg capsules (Shire Pharmaceuticals Ltd) |
| 6643 | Atomoxetine 40mg capsules |
| 11733 | Methylphenidate 20mg tablets |
| 6644 | Atomoxetine 60mg capsules |
| 6645 | Atomoxetine 25mg capsules |
| 7100 | Atomoxetine 10mg capsules |
| 13914 | Equasym 5mg tablets (Shire Pharmaceuticals Ltd) |
| 13946 | Equasym 10mg tablets (Shire Pharmaceuticals Ltd) |
| 11536 | Methylphenidate 20mg modified-release capsules |
| 35658 | Medikinet XL 30mg capsules (Flynn Pharma Ltd) |
| 35659 | Medikinet XL 20mg capsules (Flynn Pharma Ltd) |
| 13212 | Methylphenidate 10mg modified-release capsules |
| 37658 | Medikinet XL 40mg capsules (Flynn Pharma Ltd) |
| 14848 | Methylphenidate 30mg modified-release capsules |
| 35469 | Methylphenidate 27mg modified-release tablets |
| 14129 | Atomoxetine 18mg capsules |
| 36628 | Medikinet XL 10mg capsules (Flynn Pharma Ltd) |
| 17014 | Strattera 40mg capsules (Eli Lilly and Company Ltd) |
| 16949 | Strattera 25mg capsules (Eli Lilly and Company Ltd) |
| 40279 | Atomoxetine 80mg capsules |
| 17588 | Strattera 60mg capsules (Eli Lilly and Company Ltd) |
| 37237 | Medikinet 10mg tablets (Flynn Pharma Ltd) |
| 14119 | Strattera 10mg capsules (Eli Lilly and Company Ltd) |
| 37097 | Medikinet 5mg tablets (Flynn Pharma Ltd) |
| 6804 | Equasym 20mg tablets (Shire Pharmaceuticals Ltd) |
| 35515 | Methylphenidate 40mg modified-release capsules |
| 18832 | Strattera 18mg capsules (Eli Lilly and Company Ltd) |
| 13238 | Dexamfetamine 1mg/ml oral liquid |
| 46593 | Medikinet XL 5mg capsules (Flynn Pharma Ltd) |
| 36910 | Medikinet 20mg tablets (Flynn Pharma Ltd) |
| 46607 | Methylphenidate 5mg modified-release capsules |
| 41492 | Strattera 80mg capsules (Eli Lilly and Company Ltd) |
| 21399 | Equasym xl 20mg Capsule (Celltech Pharma Europe Ltd) |
| 54504 | Methylphenidate 20mg modified-release tablets |
| 24116 | Durophet 12.5mg Capsule (3M Health Care Ltd) |
| 47609 | Dexamfetamine 5mg modified-release capsules |
| 56336 | Elvanse 50mg capsules (Shire Pharmaceuticals Ltd) |
| 55987 | Lisdexamfetamine 30mg capsules |
| 31623 | Dexedrine 15mg Spansules (Imported (United States)) |
| 16185 | Dexamfetamine 15mg modified-release capsules |
| 51453 | Dexamfetamine 5mg/5ml oral solution |
| 55635 | Atomoxetine 100mg capsules |
| 18998 | Durophet 7.5mg Capsule (3M Health Care Ltd) |
| 55495 | Dexamfetamine with amfetamine 10mg with 10mg capsules |
| 55747 | Elvanse 30mg capsules (Shire Pharmaceuticals Ltd) |
| 47481 | Dexamfetamine 10mg modified-release capsules |
| 52233 | Methylphenidate 54mg modified-release tablets |
| 56742 | Elvanse 70mg capsules (Shire Pharmaceuticals Ltd) |
| 18996 | Durophet 20mg Capsule (3M Health Care Ltd) |
| 23173 | Tranquilyn 10mg tablets (Genesis Pharmaceuticals Ltd) |
| 47099 | Dexamfetamine with amfetamine 10mg with 10mg modified-release capsules |
| 49392 | Amfetamine 10mg / Dexamfetamine 10mg modified-release capsules |
| 55169 | Lisdexamfetamine 50mg capsules |
| 56576 | Elvanse 30mg capsules (Shire Pharmaceuticals Ltd) |
| 47679 | Dexamfetamine 15mg modified-release capsules |
| 23161 | Tranquilyn 5mg tablets (Genesis Pharmaceuticals Ltd) |
| 52461 | Equasym XL 10mg capsules (Waymade Healthcare Plc) |
| 54804 | Equasym XL 10mg capsules (Doncaster Pharmaceuticals Ltd) |
| 53527 | Equasym XL 30mg capsules (Waymade Healthcare Plc) |
| 56713 | Ritalin-SR 20mg tablets (Imported (United States)) |
